# Analyzing Dynamic Changes of Laboratory Indexes in Patients with Acute Heart Failure Based on Retrospective Study

**DOI:** 10.1155/2016/7496061

**Published:** 2016-04-06

**Authors:** Yurong Wang, Lei Fu, Qian Jia, Hao Yu, Pengjun Zhang, Chunyan Zhang, Xueliang Huang, Kunlun He, Yaping Tian

**Affiliations:** ^1^Core Laboratory of Translational Medicine, Chinese PLA General Hospital, Beijing 100853, China; ^2^School of Medicine, Nankai University, Tianjin 300071, China; ^3^Department of Medical Engineering, 401 Hospital of Chinese PLA, Qingdao 266071, China; ^4^Computer Section of Chinese PLA General Hospital, Beijing 100853, China; ^5^Department of Cardiology, Chinese PLA General Hospital, Beijing 100853, China

## Abstract

*Background*. Changes of N-terminal probrain natriuretic peptide (NT-proBNP) have been studied whether in the long term or the short term in patients of acute heart failure (AHF); however, changes of NT-proBNP in the first five days and their association with other factors have not been investigated.* Aims*. To describe the dynamic changes of relevant laboratory indexes in the first five days between different outcomes of AHF patients and their associations.* Methods and Results*. 284 AHF with dynamic values recorded were analyzed. Changes of NT-proBNP, troponin T, and C-reactive protein were different between patients with different outcomes, with higher values in adverse group than in control group at the same time points (*p* < 0.05). Then, prognostic use and risk stratification of NT-proBNP were assessed by receiver-operating characteristic curve and logistic regression. NT-proBNP levels at day 3 showed the best prognostic power (area under the curve = 0.730, 95% confidence interval (CI): 0.657 to 0.794) and was an independent risk factor for adverse outcome (odds ratio, OR: 2.185, 95% CI: 1.584–3.015). Classified changes of NT-proBNP may be predictive for adverse outcomes in AHF patients.* Conclusions*. Sequential monitoring of laboratory indexes within the first 5 days may be helpful for management of AHF patients.

## 1. Background

Acute heart failure (AHF) is the most common reason of hospitalization in patients aged 65 and older, with mortality rate up to 30–40% within one year [1, 2]. Prognosis is poor in hospitalized AHF patients [3, 4]. Accurate prognostic approach and risk stratification may be useful for management of AHF patients.

Surveillance of brain natriuretic peptide (BNP) and N-terminal- (NT-) proBNP in plasma has been reported to be prognostic, offering powerful risk stratification for monitoring the whole HF stages [5]. Kwan et al. concentrated on the prognostic significance of serial measurements of NT-proBNP within first 24 hours at admission and again at discharge for patients with chest pain. The results showed that dynamic surveillance of NT-proBNP in the short term did not improve its prognostic ability [6]. Lindahl et al. investigated the prognostic ability of NT-proBNP changes within six months in 1,216 patients with acute coronary syndromes [7] and found NT-proBNP level was stable from 2 days to 6 months and can be more predictive for two-year mortality. Then, Masson and his colleagues analyzed the predictive power of NT-proBNP changes at 4 months from baseline in 1,742 chronic heart failure (CHF) patients [8]. The results revealed that NT-proBNP measurements and classification into four categories of trends may be a superior approach for risk stratification for CHF patients. Myocardial injury, chronic kidney disease (CKD), and pulmonary infection are shown to be high risk factors for mortality of AHF patients, which were well characterized by troponin T (TNT), glomerular filtration rate (GFR), and C-reactive protein (CRP) [9, 10]. In 2013, a report by Metra et al. focused on the short-term (within 2 weeks) dynamics of markers for cardiac, renal, and hepatic damage and cardiac congestion in AHF patients after taking serelaxin [11]. However, sequential dynamics of NT-proBNP and other indexes during the first 5 days and relation among them have not been studied in AHF patients.

The purpose of this study was to observe the changes of NT-proBNP and other indexes within the first 5 days after AHF between patients with different outcomes and their relation, eventually to find the best prognostic approach for AHF patients.

## 2. Materials and Methods

### 2.1. Study Design and Data Collection

The present study was performed in Chinese PLA General Hospital, Beijing, China. From November 2008 to March 2015, AHF patients presenting to emergency room or intensive care unit were screened. NT-proBNP and other indexes values within the first 5 days were reviewed. Finally, 284 AHF of 251 patients with at least 3 of 5 time points were enrolled, which means that some patients have been enrolled into the study more than once. Then we recorded the baseline information, including sex, age, basic medical history, and vital signs. All data was retrieved from the electronic medical record system of the hospital. The study complied with the guidelines approved by Chinese PLA General Hospital's Ethics Committee. All subjects involved in this study provided informed consents.

The inclusion criteria were established similar to the previous study by Bian et al. [12]. Patients, aged over 18, were enrolled into the study based on the discharge diagnosis of AHF (International Classification of Diseases, Ninth/Tenth Revision coding) or combination of patients' complaint (dyspnea or orthopnea), physical examination (lung rales or oxygen desaturation), and rescue measures (oxygen, mechanical ventilation, diuretics, and cardiac stimulants) or tracheal intubation. The cases without any of the above were excluded.

Patients recovered at discharge were divided into the control group, while patients with all-cause mortality in hospital were divided into the adverse outcome group.

### 2.2. Definition

**Figure 1:**
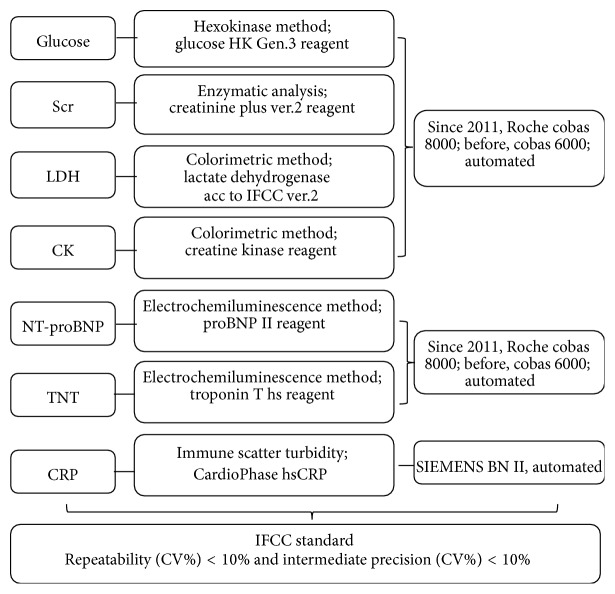
Flow chart of analytical methods of all indexes in hospital laboratory.

Analytical methods used in hospital laboratory were summarized in Figure 1. Categorization of plasma TNT, CRP, and eGFR changes was performed according to the cutoff values defined on the basic of previous researches and guidelines. In accordance with the universal definition of myocardial infarction, TNT level was defined as elevated when its value exceeded 0.01 ng/mL (the 99th percentile of the upper reference in healthy individuals) [13]. CRP was defined as elevated at the value of 1.00 mg/dL and less than 90 mL/min was defined as eGFR decline [6, 7]. The eGFR was calculated based on formula MDRD with adjustment for gender [14].

### 2.3. Statistical Analysis

Statistical analysis was performed using SPSS version 19.0. Continuous variables were presented as the mean ± standard deviation or median (25th–75th percentiles) according to normality. For differences comparison between groups, independent *t*-test was used for variables normally distributed. Mann-Whitney *U* test was applied for variables abnormal distribution. Chi-square test was used for categorical variables. Comparison for changes of NT-proBNP, CRP, TNT, and eGFR over time at the same group was assessed by the Wilcoxon signed rank test (or Friedman test). Differences in levels of NT-proBNP, CRP, TNT, and eGFR at the same time point between different groups were analyzed through the Mann-Whitney *U* test. Log-transformed NT-proBNP, TNT, and CRP were also used.

NT-proBNP changes were analyzed in 3 different ways: absolute change from basic level, relative percentage change from basic level, and tendency changes [8]. Tendency changes were defined as four categories according to a cutoff value which was calculated as NT-proBNP concentration at day 3 with the best prediction accuracy for adverse outcome: low to low (cases with NT-proBNP both below the cutoff value); high to low (cases with NT-proBNP above the value at baseline and below at day 3); low to high (cases with NT-proBNP below the value at baseline and above at day 3); and high to high (cases with NT-proBNP both both above the value).

Receiver-operating characteristic (ROC) curve was applied to assess the prognostic usage of NT-proBNP at different time points. Logistic regression was used to assess the odds ratios of single measurement of NT-proBNP and the four categories of changes for adverse outcomes. The latter one was not adjusted for other demographic or clinical indicators. In the study, two-tailed *p* value < 0.05 was defined as statistically significant.

## 3. Results

### 3.1. Clinical Baseline Characteristics

This study included 143 men and 108 women with 284 AHF recorded in total, meaning that some patients are enrolling into the study more than once. Patient baseline characteristics between different clinical outcomes are shown in Table 1. Patients with adverse outcome had more adverse baseline characteristics, such as older age (median 74 years; Q1–Q3, 61–80 years), higher prevalence of CKD (55.2%) and stroke (29.2%), and higher level of NT-proBNP, TNT, and CRP in plasma, while in controls, NYHA classes III to IV (80.9%) and a history of CAD (52.1%) were more prevalent.

### 3.2. Dynamic Changes of Laboratory Indexes

**Figure 2:**
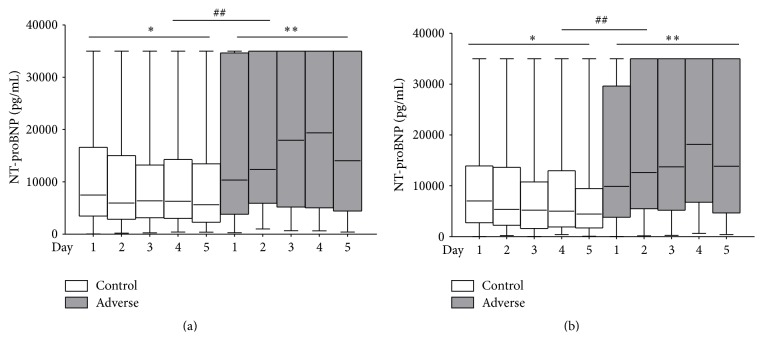
Plasma NT-proBNP changes over time in AHF patients. (a) showed NT-proBNP levels at days 1 (baseline), 2, 3, 4, and 5 in 85 AHF with measurements at all time points (control, *p* = 0.031 and adverse, *p* = 0.003). (b) NT-proBNP levels over time in all 284 AHF with measurements of 284, 237, 211, 185, and 180 at days 1, 2, 3, 4, and 5, respectively (control, *p* = 0.018, and adverse, *p* = 0.008). ^*∗*^ *p* < 0.05, ^*∗∗*^ *p* < 0.01 (significance in the same group); ^##^ *p* < 0.01 (significance between different groups).

Significant differences were observed in baseline of NT-proBNP, TNT, CRP, and eGFR between groups with different outcomes (Table 1). Then we analyzed the dynamic changes of the four indexes. Firstly, in 85 AHF with NT-proBNP measurements at all five time points, dynamics of NT-proBNP had a significant decrease over time in control group (*p* = 0.031) but an observable elevation in adverse outcome group (Figure 2(a), *p* = 0.003). Relatively consistent results were obtained in all AHF with NT-proBNP examinations not less than 3 of 5 time points (Figure 2(b), control: *p* = 0.008; adverse: *p* = 0.018). In control group, NT-proBNP baseline was 7024 pg/mL (2748–13923 pg/mL) and decreased to 4438 pg/mL (1725–9460 pg/mL) at day 5; however, in adverse group, NT-proBNP concentration was 9922 pg/mL (3825–29632 pg/mL) at baseline and increased to 13855 pg/mL (4670–35000 pg/mL) at day 5. Then changes of other indexes in all patients with at least 3 time points were described as below.

**Figure 3:**
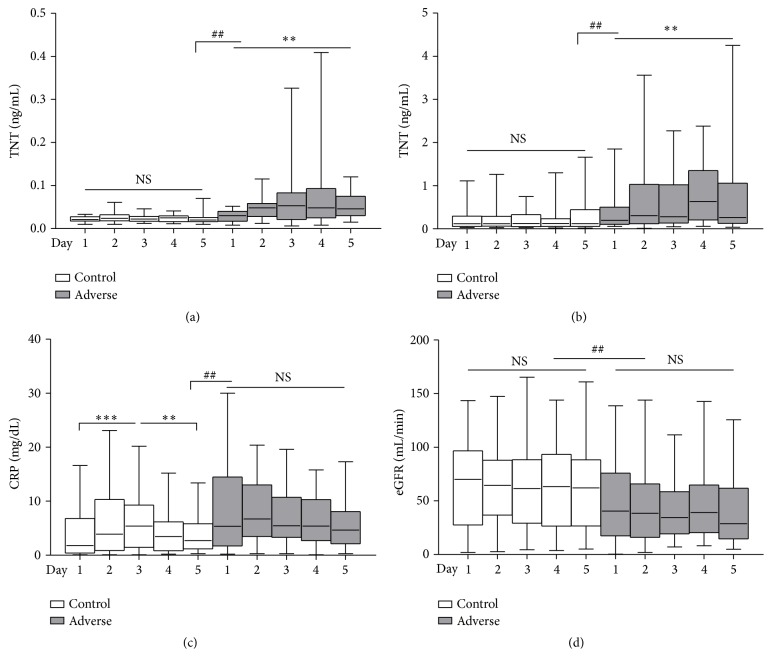
Changes of TNT, CRP, and eGFR over time between AHF patients with different outcomes. TNT changes categorized by percentile at baseline: (a) the first tertile: control, *p* > 0.05, and adverse, *p* = 0.007; (b) the upper two tertiles: control, *p* > 0.05, and adverse, *p* = 0.005; (c) changes of CRP over time: control, days 1 to 3, *p* < 0.001, days 3 to 5, *p* = 0.003, and adverse, *p* > 0.05; and (d) changes of eGFR over time: control, *p* > 0.05, and adverse *p* > 0.05. ^*∗∗∗*^ *p* < 0.001, ^*∗∗*^ *p* < 0.01 (significance in the same group); ^##^ *p* < 0.01 (significance between different groups). NS: not significant.

For TNT, in the first tertile, the baseline in control group was 0.021 ng/mL (0.018–0.028 ng/mL) and 0.020 ng/mL (0.016–0.026 ng/mL) at day 5 (*p* > 0.05); however, in adverse group, it was 0.030 ng/mL (0.017–0.040 ng/mL) at baseline and increased to 0.046 ng/mL (0.030–0.075 ng/mL) at day 5 (Figure 3(a), *p* = 0.007). Then in the upper two tertiles, TNT median (Q1–Q3) level at baseline was 0.117 ng/mL (0.056–0.293 ng/mL) and 0.120 ng/mL (0.054–0.442 ng/mL) in control group, but in adverse group, TNT baseline was 0.196 ng/mL (0.104–0.503 ng/mL) and elevated to 0.265 ng/mL (0.126–1.058 ng/mL) at day 5 (Figure 3(b), *p* = 0.005). As shown, TNT remained at low level over time in control group but continued to rise in adverse group (*p* < 0.01).

For patients in control group, CRP median (Q1–Q3) level was 1.80 mg/dL (0.40–6.80 mg/dL) at admission and increased to 3.90 mg/dL (0.88–10.33 mg/dL) at day 3 and then dropped to 2.70 mg/dL (1.18–5.83 mg/dL) at day 5; however, in adverse group, CRP baseline was 5.35 mg/dL (1.73–14.48 mg/dL) and 5.45 mg/dL (3.30–10.73 mg/dL) at day 3 and 4.65 mg/dL (2.13–8.08 mg/dL) at day 5. So, CRP underwent a process of first rose (*p* < 0.001) and then descended (*p* = 0.003) but kept at high level in adverse group without a significant decrease (Figure 3(c)).

In adverse group, eGFR maintained a lower level than that in control group (Figure 3(d)). Baseline of eGFR was 70 mL/min (27–97 mL/min) without a significant decrease over time in control group; however, in adverse group, it was 41 mL/min (17–76 mL/min) at admission and decreased to 29 mL/min (15–62 mL/min) at day 5. Besides, median levels of the four indexes were significantly different between different groups at the same time points (*p* < 0.01) except that CRP level at day 3.

### 3.3. Baseline and Changes of NT-proBNP Related to Other Indexes

**Figure 4:**
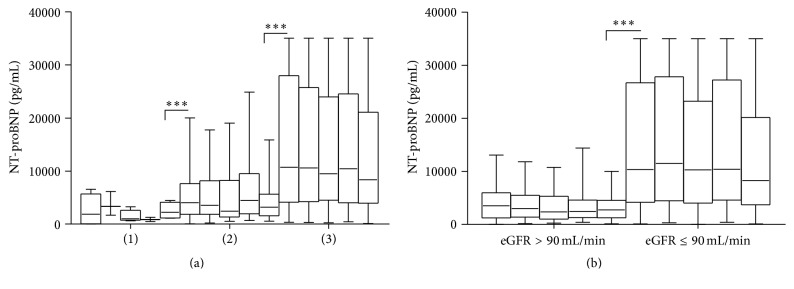
Changes of NT-proBNP level at admission, days 2, 3, 4, and 5 in patients: (a) (1) without TNT elevation: TNT < 0.01 ng/mL; (2) with TNT elevation, but without elevated CRP: TNT⩾0.01 ng/mL and CRP < 1.00 mg/dL; (3) with elevation of both: TNT⩾0.01 ng/mL and CRP⩾1.00 mg/dL. (b) With eGFR decline and without. ^*∗∗∗*^ *p* < 0.001.

Univariate analysis showed that NT-proBNP baseline was related to age, TNT, CRP, and eGFR and not significantly associated with history of CAD and stroke, NYHA functional classification, and DBP (Table 2). TNT and CRP baseline level positively correlated with NT-proBNP levels at any time points (Figure 4(a), *p* < 0.001). And NT-proBNP concentration was elevated at all time points when baseline eGFR declined below 90 mL/min (Figure 4(b)).

### 3.4. NT-proBNP Changes for Predicting the Adverse Outcome of AHF Patients

Because NT-proBNP changes within the first 3 days were stable and more predictive for adverse outcomes, we compared the predictive power of single measurement of NT-proBNP (at days 1, 2, and 3) versus dynamic changes, indicated as absolute or relative changes. ROC curve was used to evaluate the adverse outcome and AUC (95% CI) increased over time (at admission, days 2 and 3), which was 0.608 (0.532 to 0.681), 0.695 (0.621 to 0.762), and 0.730 (0.657 to 0.794), respectively. And the cutoff value of NT-proBNP was 8459 pg/mL at day 3. The absolute or relative changes have not improved the prognostic power with 0.680 (0.605 to 0.748) and 0.686 (0.612 to 0.754). In logistic regression model, NT-proBNP concentration at day 3 was an independent predictor for the adverse outcome (OR 2.185, 95% CI: 1.584–3.015), along with advanced age and history of CAD and CRP (Table 3). Variables associated with the adverse outcome with *p* < 0.05 entered as confounding factors in multivariate analysis.

### 3.5. Classified Changes of NT-proBNP

**Figure 5:**
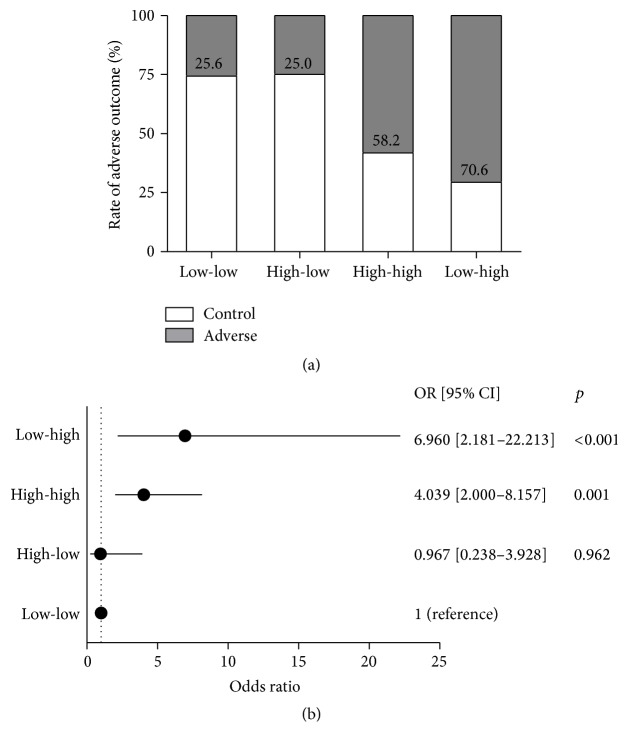
Classified changes of NT-proBNP. (a) Percentage and (b) odds ratio of classified changes of NT-proBNP for the adverse outcome of AHF patients.

Adverse outcome rates for AHF patients classified by categorical changes of NT-proBNP were presented in Figure 5(a). Adverse outcome rate of patients in high to low group (threshold of 8459 pg/mL) was close to those of low to low group (25.0% versus 25.6%) and higher in patients of low to high group than those in group of high to high (70.6% versus 58.2%).

Patients with decreased level of NT-proBNP (high to low) had a risk for adverse outcome with OR of 0.967, 95% CI: 0.238–3.928, similar to those in the reference group (Figure 5(b)). In contrast, patients with worsened level of NT-proBNP had an obviously increased risk of adverse outcome (low to high, OR: 6.960, 95% CI: 2.181–22.213, *p* < 0.001), higher than those remaining at high level (high to high, OR: 4.039, 95% CI: 2.000–8.157, *p* = 0.001). The percentage of patients taking ACEI/ARB and beta-blockers was higher in control group than that in adverse group (45.2% versus 17.9%; 55.9% versus 38.9%). And the proportion of patients taking vasoactive drugs and morphine was lower in control group than that in adverse group (33.5% versus 61.1%; 13.8% versus 34.7%). It suggests that ACEI/ARB and beta-blockers would have protective activity in short-term management of AHF patients (Table 4).

## 4. Discussion

Acute heart failure is characterized by the acute episodes of signs and symptoms because of decompensation of cardiac function, needing emergency treatment. Mortality rate in hospital reaches up to 10% [15]. NT-proBNP is the well-established marker for HF, and its baseline level is predictive for patients with adverse outcomes in short-term [16, 17]. Lindahl and his colleagues found that serial measurements of NT-proBNP could improve its prognostic utility with increasing odds ratio over time [7]. Although the changes in laboratory parameters are important within hours of AHF, it is also necessary to analyze them within the first few days. The purpose of our study is to describe dynamic trends of several main indexes related to outcome of AHF, to warn us to pay close attention on clinical management in the future, and finally to decrease the mortality. In the present study, NT-proBNP level at day 3 had the best predictive accuracy within the first 3 days, suggesting that dynamic monitoring NT-proBNP is instructive for AHF management. Moreover, classified changes of NT-proBNP at day 3 from baseline would improve the predictive value of its single determination in patients. And this stratification strategy is also used in a different setting of 116 AHF patients, in which NT-proBNP was tested at different time points with intervals from the beginning of treatment to discharge [18]. And their results showed that absolute changes at discharge from baseline had the best predictive power, similar to conclusions from other investigations [19]. Our analysis showed that the usage of stratification strategy in NT-proBNP was more helpful for management of AHF patients.

As shown, higher level of TNT at baseline was associated with higher baseline and elevation of NT-proBNP over time. We know that plasma TNT level is well established for MI [20]. Elevated TNT level may be due to ongoing of the established myocardial necrosis or large infarction area [21, 22]. Both of them could result in the dysfunction of left ventricular and then the subsequent NT-proBNP elevation at baseline. In the present study, TNT remained at low level (baseline median = 0.052 ng/mL) in control group; however, it worsened over time (baseline median = 0.104 ng/mL) in patients with adverse outcome. In addition, NT-proBNP and TNT sharing the similar trends in adverse group may illustrate the reason above.

More and more researches showed that CRP may be involved in HF development through activating complement system, stimulating cytokine generation, and then resulting in myocyte loss and functional deterioration [23–25]. Our results showed that CRP in control group rose at first and then descended and the level overall was lower than that in adverse group. Moreover, CRP level had an influence on NT-proBNP because elevation of baseline CRP caused elevation of NT-proBNP at baseline and all the time points. Tumor necrosis factor-alpha and interleukin-6 were reported to associate with myocardial dysfunction, then mediating the expression of BNP and NT-proBNP [26, 27]. CRP can also be upregulated by interleukin-6. Therefore, it could explain the association between CRP level and NT-proBNP changes.

The mechanism of ACEIs and ARBs on renin angiotensin system (RAS) is different. ACEI inhibits the angiotensin-converting enzyme and ARBs inhibits Ang II activation through competitively binding to AT1 receptor. Consequently, vascular resistance is reduced, aldosterone is released, and salt and water retention is prevented, causing reduced cardiac preload and afterload. As reported, ARB drugs gave limited end-points benefits to patients with myocardial infarction and cardiovascular disease, compared with placebo [28, 29]. However, other investigations showed that ACEI/ARB and beta-blockers drugs can significantly reduce all-cause mortality and cardiovascular mortality [30–33]. Consistent with them, our results showed that in control group the proportion of patients who received ACEI/ARB drugs and beta-blockers was significantly higher than that in adverse group, further determining their protective roles in management of AHF patients. Morphine use was considered to be associated with increased in-hospital death of AHF patients [34, 35]. Besides, a review from Ruiz-Laiglesia and Camafort-Babkowski suggested that vasoactive drugs may give benefits in the short term but cause mortality in the long term [36]. Combined with our results, morphine and vasoactive drugs should be used with caution in AHF.

## 5. Study Limitations

This study is a single-center and retrospective control study. Collecting complete continuous data was difficult. Only 85 AHF had complete data sets at all 5 time points, 176 AHF with all first 3 time points, and 284 AHF with at least 3 of 5 time points. Many patients were excluded from this research for without baseline or with only one measurement or died within 24 hours. Second, as a retrospective investigation, we are not sure whether specific treatment influences the outcomes. Finally, sequential monitoring of lab indexes for AHF needs to be assessed and validated in multicenter and large sample prospective research.

## 6. Conclusions

In this single-site study with relative small samples, the prognostic value was better in dynamic assessment of NT-proBNP than baseline level. Sequential monitoring of laboratory indexes within the first 3 days of AHF may be helpful for guiding clinical management of AHF patients.

## Figures and Tables

**Table 1:** Comparisons of baseline characteristics between AHF patients with different clinical outcomes.

*N* = 284			
	Control (*n* = 188)	Adverse event (*n* = 96)	*p* value
Age (years), median (Q1–Q3)	67 (52–77)	74 (61–80)	0.006
Male, *n* (%)	109 (58.0)	55 (57.3)	0.912
NYHA III to IV, *n* (%)	152 (80.9)	67 (69.8)	0.036
History			
CAD, *n* (%)	98 (52.1)	67 (35.6)	0.004
MI, *n* (%)	77 (41.0)	39 (40.6)	0.957
Hypertension, *n* (%)	133 (70.7)	68 (70.8)	0.988
Diabetes, *n* (%)	72 (38.3)	40 (41.7)	0.583
Arrhythmia, *n* (%)	64 (34.0)	36 (37.5)	0.564
COPD, *n* (%)	37 (19.7)	20 (20.8)	0.819
CKD, *n* (%)	64 (34.0)	53 (55.2)	0.001
Stroke, *n* (%)	24 (12.8)	28 (29.2)	0.001
Vital signs			
RR (bpm), median (Q1–Q3)	18 (18–20)	18 (18–21)	0.357
Pulse (bpm), mean ± SD	94 ± 21	95 ± 23	0.656
SBP (mmHg), mean ± SD	132 ± 27	126 ± 23	0.076
DBP (mmHg), mean ± SD	78 ± 17	72 ± 14	0.005
Lab indexes			
LVEF (%), mean ± SD	43 ± 11	47 ± 13	0.114
NT-proBNP (pg/mL), median (Q1–Q3)	7024 (2729–13923)	9922 (3825–29632)	0.017
TNT (ng/mL), median (Q1–Q3)	0.052 (0.026–0.194)	0.104 (0.042–0.447)	0.002
CRP (mg/dL), median (Q1–Q3)	1.69 (0.40–5.77)	4.20 (1.40–14.00)	<0.001
eGFR (mL/min), median (Q1–Q3)	73 (32–98)	44 (21–80)	0.004
CK (U/L), median (Q1–Q3)	98.9 (53.8–171.1)	87 (46.8–169.5)	0.251
LDH (U/L), median (Q1–Q3)	259.5 (209.6–334.2)	286.8 (220.1–443.5)	0.077
Glucose (mmol/L), median (Q1–Q3)	8.88 (6.25–12.23)	8.06 (6.13–10.54)	0.179

AHF: acute heart failure; CAD: coronary artery disease; CK: creatine kinase; CKD: chronic kidney disease; CK-MB: creatine kinase isoenzyme; COPD: chronic obstructive pulmonary disease; CRP: C-reactive protein; DBP: diastolic blood pressure; eGFR: estimated glomerular filtration rate; LDH: lactic dehydrogenase; LVEF: left ventricular ejection fraction; MI: myocardial infarction; NT-proBNP: N-terminal probrain natriuretic peptide; NYHA: New York Heart Association; Q: quartile; RR: respiratory rate; SBP: systolic blood pressure; and TNT: troponin T.

**Table 2:** NT-proBNP baseline related to baseline factors.

	Median pg/mL (Q1–Q3)	*p* value
Age		
<65 years	7994 (3320–19961)	0.011
<75 years	10800 (4160–28062)	
<85 years	5319 (2238–10091)	
≥85 years	7689 (3588–14203)	
NYHA		
I to II	7689 (2323–16930)	0.578
III to IV	7733 (3375–18439)	
CAD		
No	8684 (3889–19139)	0.094
Yes	7253 (2580–13827)	
Stroke		
No	7602 (3074–17651)	0.710
Yes	8263 (3319–19665)	
DBP		
<78 mmHg	7295 (2932–17520)	0.164
≥78 mmHg	7948 (3320–19791)	
TNT		
<0.01 ng/mL	1270 (31–4474)	0.003
≥0.01 ng/mL	7744 (3214–17818)	
CRP		
<1.00 mg/dL	5105 (2007–9551)	<0.001
≥1.00 mg/dL	10437 (3921–27245)	
eGFR		
≥90 (mL/min)	4075 (1505–7492)	<0.001
<90 (mL/min)	10158 (4170–26902)	

Abbreviations are the same as shown in Table 1.

**Table 3:** Significant predictors for adverse outcome among AHF patients.

	OR	95% CI	*p* value
Age^*∗*^ (65 years, +10 years)	2.032	1.147–3.601	0.015
History of CAD	4.448	1.524–12.979	0.006
Baseline CRP (1 increment on log scale)	1.856	1.420–2.425	<0.001
NT-proBNP at day 3 (1 increment on log scale)	2.905	1.583–5.331	0.001

^*∗*^Age: centered at 65 years and 1 increment on 10-year scale.

**Table 4:** Management of AHF patients between different clinical outcomes.

Emergency treatment at admission	Control	Adverse outcome	*p* value
Diuretics, *n* (%)	169 (89.9)	87 (91.6)	0.649
ACEI/ARB, *n* (%)	85 (45.2)	17 (17.9)	<0.001
Beta-blockers, *n* (%)	105 (55.9)	37 (38.9)	0.007
Vasoactive drugs, *n* (%)	63 (33.5)	58 (61.1)	<0.001
Morphine, *n* (%)	26 (13.8)	33 (34.7)	<0.001
Digoxin, *n* (%)	78 (41.5)	39 (41.1)	0.944
Nitrates, *n* (%)	120 (63.8)	55 (57.9)	0.332
Oxygen supply, *n* (%)	168 (89.4)	86 (90.5)	0.760
Antiasthmatic, *n* (%)	111 (59.0)	58 (61.1)	0.745

ACEi: angiotensin-converting enzyme inhibitor; ARB: angiotensin receptor blocker.

## Abbreviations

ACEi: Angiotensin-converting enzyme inhibitor
AHF: Acute heart failure
ARB: Angiotensin receptor blocker
CAD: Coronary artery disease
CHF: Chronic heart failure
CI: Confidential interval
CK: Creatine kinase
CKD: Chronic kidney disease
CK-MB: Creatine kinase isoenzyme
COPD: Chronic obstructive pulmonary disease
CRP: C-Reactive protein
DBP: Diastolic blood pressure
eGFR: Estimated glomerular filtration rate
LDH: Lactic dehydrogenase
LVEF: Left ventricular ejection fraction
MI: Myocardial infarction
NT-proBNP: N-Terminal probrain natriuretic peptide
OR: Odds ratio
NYHA: New York Heart Association
Q: Quartile
ROC: Receiver-operating characteristic curve
RR: Respiratory rate
SBP: Systolic blood pressure
TNT: Troponin T.
